# Supplementary material for: Network controllability of structural connectomes in the neonatal brain
Source: Nat Commun. 2023 Sep 19;14:5820. doi: 10.1038/s41467-023-41499-w (PMC10509217; doi:10.1038/s41467-023-41499-w)
Supplement: Supplementary file 1 — Supplementary information [file 41467_2023_41499_MOESM1_ESM.pdf]

Supplementary information

**Fig. S1 Analysis with modal controllability**	**3**
**Table S1: Top 10 brain regions with highest controllability**	**4**
**Fig. S2: Control roles of cognitive networks.**	**6**
**Fig. S3: Regions with the longitudinal difference in average controllability maturation during perinatal period.**	**8**
**Fig. S4: Regional controllability development rate for preterm and term infants.**	**9**
**Table S2: Structural network properties for each infant group (mean(std)) and their associations with controllability**	**11**
**Table S3: Controllability maturation during perinatal period controlling for network strength/network density (r-value between controllability and postmenstrual age)**	**12**
**Table S4: Pearson's correlation between regional controllability value and its maturation controlling for network strength/network density**	**13**
**Fig. S5: Group differences in controllability between preterm and term infants with network strength as the additional covariance.**	**14**
**Fig. S6: Group differences in controllability between preterm and term infants with network density as the additional covariance.**	**15**
**Table S5: Sex consistency in the whole-brain controllability (Spearman's correlation between whole-brain controllability in female and male groups)**	**17**
**Table S6: Sex difference in controllability maturation (Pearson's correlation between controllability and post-menstrual age)**	**18**
**Table S7: Control energy consumption for null models and real brain networks of term and preterm infants (t-stats from paired t-test)**	**19**
**Fig. S7: Scatterplots of controllability at birth and behavioral performances at 18 months old.**	**20**
**Table S8. Neonatal 90 node parcellation's regional names and resting-state networks(RSNs)**	**21**
**Supplementary reference**	**23**

Analysis with modal controllability

Average controllability and modal controllability are two distinct but complementary measurements of network controllability based on the network control theory. While average controllability quantifies the ability of each node to drive nearby state transitions with minimal energy input, modal controllability measures the capacity to drive more distant state transitions with higher energy requirements. Although these two measurements are highly correlated, they exhibit differences in several ways. In the main text, we presented the analysis of average controllability, while in this supplementary section, we discussed modal controllability to provide a comprehensive understanding of the controllability of the infant brain network.

Regional average controllability was negatively correlated with regional modal controllability average across all three infant groups ($r<-0.99$, $p<0.001$), in alignment with the structural connectomes of older individuals [1]. The strongest regions with modal controllability were mainly concentrated in somatomotor, dorsal attention, frontoparietal and subcortical networks. For modal controllability, the important cognitive networks in the infant brain overlap with the adult brain, mainly including the somatomotor and frontoparietal networks (Fig. S1a).

In contrast to whole-brain average controllability, whole-brain modal controllability changed similarly between 38-44 weeks PMA ($r=-0.35$, $p=0.002$) compared to 28-36 weeks PMA ($r=-0.44$,$p=9.5e-5$) for preterm infants. For the term group, postmenstrual age at scan negatively correlated with whole-brain modal controllability $r=-0.50$, $p=2.7e-29$; Fig. S1b). No significant difference in the rate of maturation on the regional level between term infants and preterm infants at TEA was detected.

For modal controllability, term and preterm infants showed no significant whole-brain level difference ($t=-0.46$, $p=0.64$, $df=519$). The patterns of group differences in regional controllability were reversed to that of average controllability and the affected regions by preterm birth were more widely distributed than those of average controllability (Fig. S1c). Most significantly changing regions are located in the frontal cortex and limbic lobe, where preterm infants change more rapidly compared to term infants (Fig. S1d).

Similar associations were observed for modal controllability (term: $r=0.21$, $p=0.047$; preterm at TEA: $r=0.37$, $p=3.2e-4$), but the difference between preterm and term groups was insignificant ($z=1.36$, $p=0.086$) (Fig. S1e).

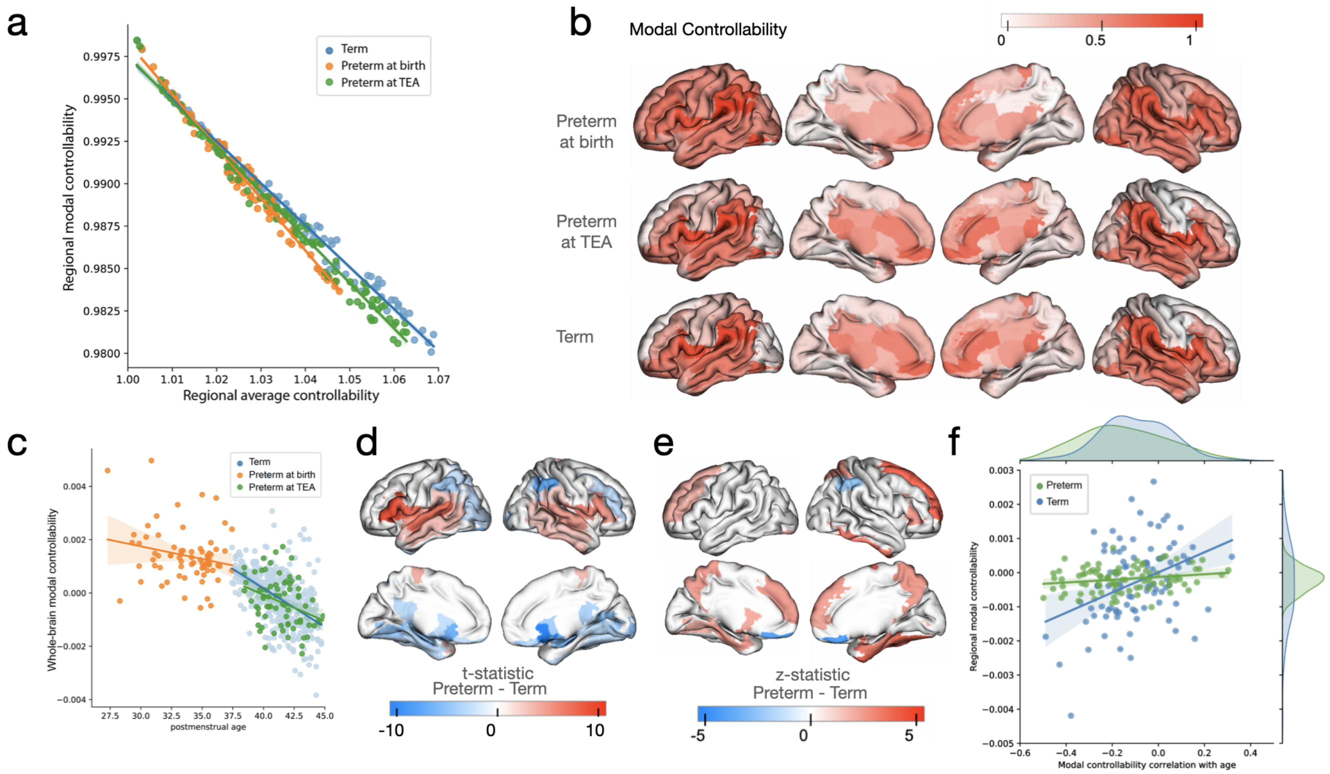

Fig. S1 Analysis with modal controllability

(a) Negative associations between regional (or, the mean controllability across every individual for each brain region) average controllability and regional modal controllability among three subgroups (Pearson's correlation: term r=-0.99, p=1.8e-82; preterm at birth r=-0.99, p=1.0e-78;preterm at TEA r=-0.99, p=6.3e-84; two-sided). Each dot represents the regional average and modal controllability for each brain region.(b) Normalized regional modal controllability was spatially similar across the preterm at birth, preterm at TEA infants, and term groups. (c) Modal controllability matures steadily over the perinatal period (Pearson's correlation: preterm at birth: r=-0.44,p=9.5e-5; preterm at TEA: r=-0.35,p=0.0024; term: r=-0.50, p=2.7e-29; two-sided). The shaded envelope denotes the 95% confidence interval. The gradual change in normalized modal controllability is shown underneath the timeline on the brain maps. (d) Modal controllability distribution differences between preterm and term infants (t stats value from the two-sampled t-test). (e) Modal controllability maturation rate differences between preterm and term infants (z-stats value from correlation comparison). (f) Positive correlations between regional modal controllability and their rate of maturation for both preterm (Pearson's r=0.37, p=3.2e-4; two-sided) and term infants(Pearson's r=0.21, p=0.047; two-sided). The shaded envelopes in the scatterplots denote the 95% confidence interval. Source data are provided as a Source Data file.

Regions and networks with the highest controllability.

Table S1: Top 10 brain regions with highest controllability

**Average Controllability**			**Modal Controllability**		
Region	Lobe	Network	Region	Lobe	Network
Preterm at birth					
Precuneus left	Parietal	DM	Heschl gyrus left	Temporal	SM
Lingual gyrus left	Occipital	Visual	Heschl gyrus right	Temporal	SM
Precuneus right	Parietal	DA	Supramarginal gyrus left	Parietal	SM
Calcarine cortex left	Occipital	Visual	Angular gyrus left	Parietal	DA
Lingual gyrus right	Occipital	Visual	Inferior occipital gyrus left	Occipital	DA
ParaHippocampal gyrus right	Limbic	Sub	Inferior frontal gyrus (opercular) left	Frontal	FP
Middle cingulate gyrus right	Limbic	VA	Rolandic operculum left	Temporal	SM
ParaHippocampal gyrus left	Limbic	Sub	Supramarginal gyrus right	Parietal	SM
Calcarine cortex right	Occipital	Visual	Inferior parietal lobule left	Parietal	DA
Cuneus	Occipital	Visual	Inferior parietal lobule right	Parietal	DA
Preterm at TEA					
Superior frontal gyrus	Frontal	DA	Heschl gyrus right	Temporal	SM
Precuneus left	Parietal	DM	Heschl gyrus left	Temporal	SM
Lingual gyrus left	Occipital	Visual	Supramarginal gyrus left	Parietal	SM
Precentral gyrus right	Central	SM	Pallidum left	Deep grey matter	Sub
Lingual gyrus right	Occipital	Visual	Rolandic operculum left	Temporal	SM
Superior frontal gyrus (dorsal) left	Frontal	DA	Pallidum right	Deep grey matter	Sub

Superior occipital gyrus left	Occipital	Visual	Inferior frontal gyrus (opercular) left	Frontal	FP
ParaHippocampal gyrus right	Limbic	Sub	Rolandic operculum right	Temporal	SM
Precuneus right	Parietal	DA	Supramarginal gyrus right	Parietal	SM
Superior parietal gyrus right	Parietal	DA	Angular gyrus left	Parietal	DA
Term					
Precuneus left	Parietal	DM	Heschl gyrus right	Temporal	SM
Precentral gyrus right	Central	SM	Heschl gyrus left	Temporal	SM
Paracentral lobule left	Central	SM	Inferior parietal lobule right	Parietal	DA
Superior frontal gyrus (dorsal) right	Frontal	DA	Supramarginal gyrus left	Parietal	SM
Superior occipital gyrus left	Occipital	Visual	Pallidum left	Deep grey matter	Sub
Supplementary motor area left	Central	DA	Angular gyrus left	Parietal	DA
Superior frontal gyrus (dorsal) left	Frontal	DA	Pallidum right	Deep grey matter	Sub
ParaHippocampal gyrus right	Limbic	Sub	Inferior parietal lobule left	Parietal	DA
Lingual gyrus right	Occipital	Visual	Supramarginal gyrus right	Parietal	SM
Lingual gyrus left	Occipital	Visual	Rolandic operculum left	Temporal	SM

The network affiliation was based on the Yeo networks : 1. Visual, 2. Somatomotor (SM), 3. Dorsal Attention (DA), 4. Ventral Attention (VA), 5. Limbic, 6. Frontoparietal (FP), 7. Default (DM), 8. Subcortical (Sub).

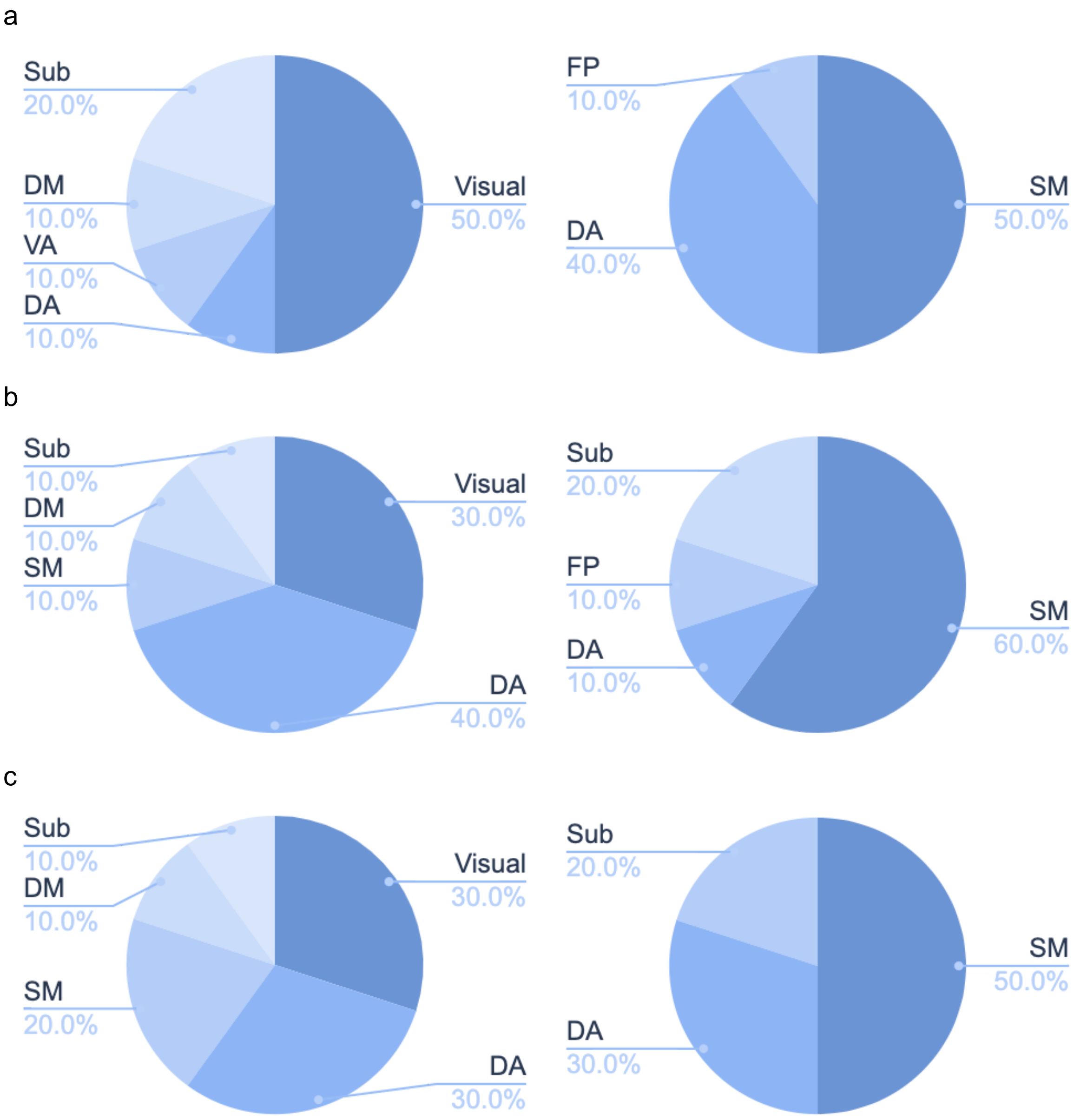

Fig. S2: Control roles of cognitive networks.

We calculated the role of each cognitive network in controllability as the percentage of regions with the highest controllability located in each network. (a) For preterm infants at birth, the visual network plays the dominant role in average controllability, and the somatomotor and dorsal attention networks are dominant in modal controllability. (b) For preterm infants at TEA, most regions with high average controllability are located in visual and dorsal attention networks and those with high modal controllability are located in the somatomotor system. (c) For term infants, regions with high average controllability are concentrated in visual, dorsal attention and somatomotor networks; regions with high modal controllability are concentrated in somatomotor, dorsal attention and subcortical networks. The network affiliation was based on the Yeo networks: 1. Visual, 2. Somatomotor (SM), 3. Dorsal Attention (DA), 4. Ventral Attention (VA), 5. Limbic, 6. Frontoparietal (FP), 7. Default (DM), 8. Subcortical (Sub).

Longitudinal difference in average controllability maturation during perinatal period.

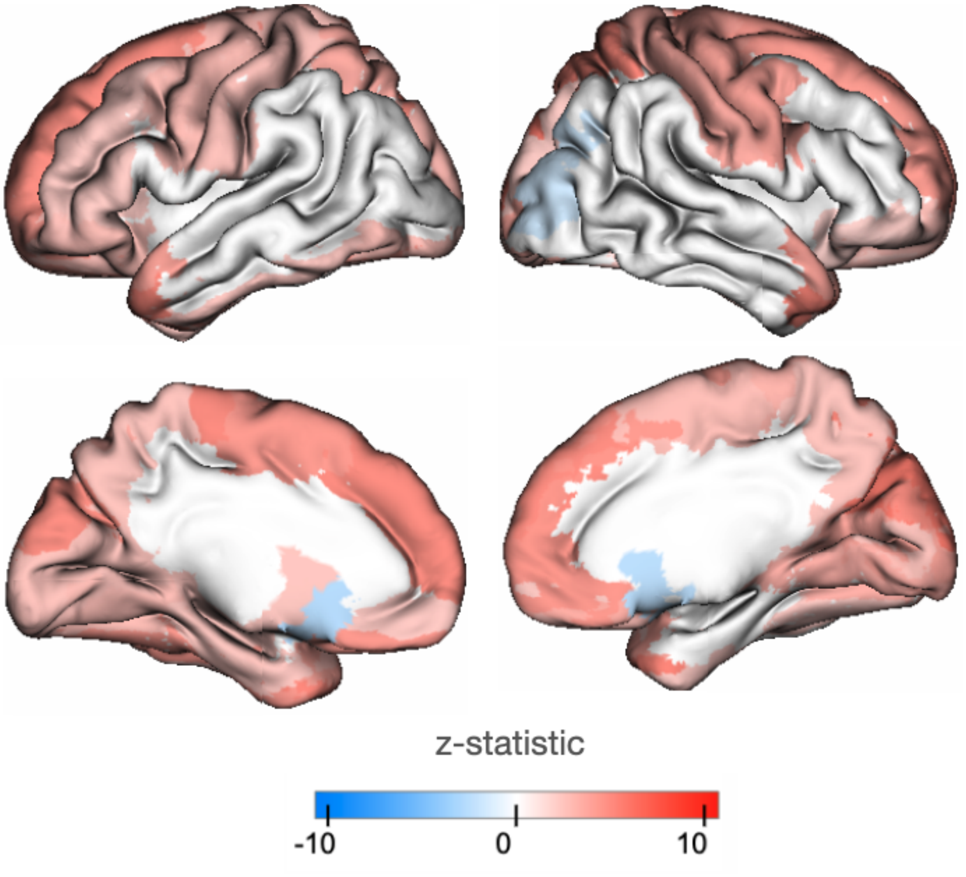

Fig. S3: Regions with the longitudinal difference in average controllability maturation during perinatal period.

Regions with significant longitudinal differences for preterm infants (at birth v.s. at TEA) in average controllability maturation (z test for comparison of correlations). Source data are provided as a Source Data file.

Regional controllability maturation rate for preterm and term infant

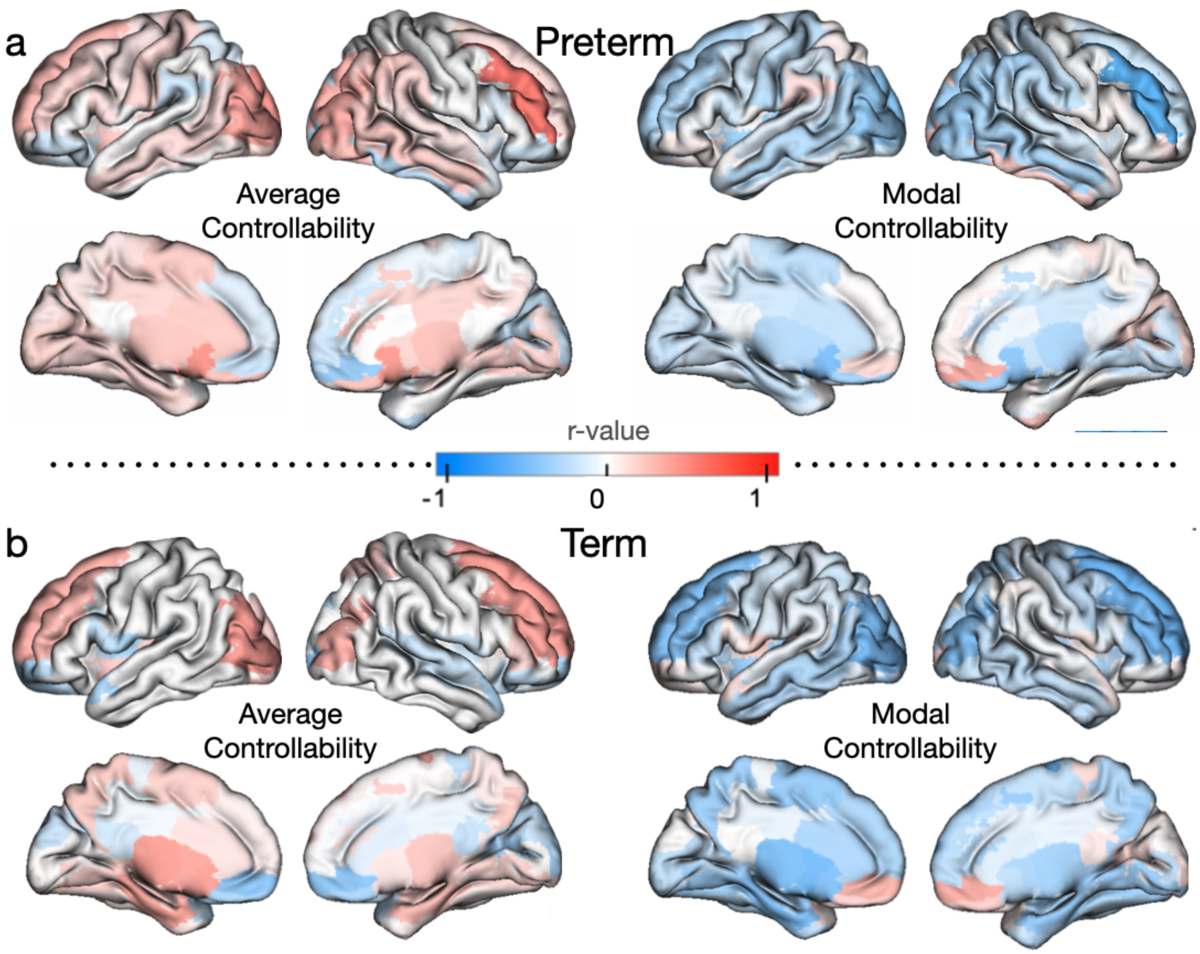

Fig. S4: Regional controllability development rate for preterm and term infants.

The r-values from Pearson's correlation between regional controllability and post menstrual age are shown for **(a)** preterm and **(b)** term infants on the brain maps. Source data are provided as a Source Data file.

Controlling for network measurements

Considering that regional connectivity was correlated with controllability, one may ask if these network properties such as network strength and network density influence our results.

First, we incorporated network strength as an additional covariate in our analysis of whole-brain and regional controllability development. Our findings indicate that the correlations between controllability values and postmenstrual age for term and preterm infants are consistent with the developmental trends we showed in the main text. Second, we also verify that network density can not explain our results by including network density as covariance in our calculations. Average controllability remains positively correlated with postmenstrual age and modal controllability continues to be negatively correlated with postmenstrual age (Table S3). Furthermore, the results that regions with higher controllability value increase more with age are still present for term and preterm infants after controlling network strength or network density (Table S4).

Furthermore, we repeated the analysis of the effects of preterm birth on controllability distribution and controllability maturation with network strength (Fig. S5) and network density (Fig. S6) as additional covariances separately. Overall, the preterm-birth influence patterns were similar to the results shown in the main text (Fig. 4).

Additionally, to test if NCT reflects a unique topology of the infant brain, we constructed null models that preserved the degree distribution on the real brain data and then repeated the controllability analysis. After permuting connection, brain networks for over 97% infants showed higher average and modal controllability compared to permuted brain networks.

In all, these results suggest that network strength and density do not provide a convincing explanation for our findings about controllability and its development in the structural brain networks during the early stage of infants' life.

Table S2: Structural network properties for each infant group (mean(std)) and their associations with controllability

Group	Preterm		Term
**Scan**	**At birth**	**At TEA**	
**Network Strength**	0.045(0.015)	0.060(0.0088)	0.065(0.011)
**Network Density**	0.53(0.15)	0.55(0.070)	0.57(0.069)
Pearson's Correlation (r, p)			
**Corr(Network Strength, AC)**	(0.88,9.5e-25)	(0.86,5.5e-22)	(0.91,2.4e-169)
**Corr(Network Strength, MC)**	(-0.67,7.0e-11)	(0.0019,0.98)	(-0.13,0.0045)
**Corr(Network Density, AC)**	(0.54,8.3e-7)	(0.12,0.33)	(0.21,8.2e-6)
**Corr(Network Density, MC)**	(-0.36,0.0016)	(0.78,3.8e-16)	(0.69,8.7e-66)

Table S3: Controllability development during perinatal period controlling for network strength/network density (r-value between controllability and postmenstrual age)

	Average Controllability		Modal Controllability	
Controlling for Network Strength				
	r	*p*	*r*	*p*
Preterm at birth	0.59	5.1e-8	-0.14	0.23
Preterm at TEA	0.37	0.0013	-0.35	0.0024
Term	0.54	1.8e-34	-0.53	2.4e-34
Controlling for Network Density				
	r	*p*	*r*	*p*
Preterm at birth	0.087	0.46	-0.23	0.051
Preterm at TEA	0.20	0.091	-0.16	0.19
Term	0.21	8.7e-6	-0.21	1.2e-5

Table S4: Pearson's correlation between regional controllability value and its development controlling for network strength/network density

	Average Controllability		Modal Controllability	
Controlling for Network Strength				
	r	*p*	*r*	*p*
Preterm at TEA	0.42	3.9e-5	0.43	2.9e-5
Term	0.14	0.18	0.067	0.53
z-stats	2.39	0.0085	3.06	0.0012
Controlling for Network Density				
	r	*p*	*r*	*p*
Preterm at TEA	0.35	7.2e-4	0.35	6.6e-4
Term	0.18	0.090	0.080	0.44
z-stats	1.52	0.065	2.22	0.013

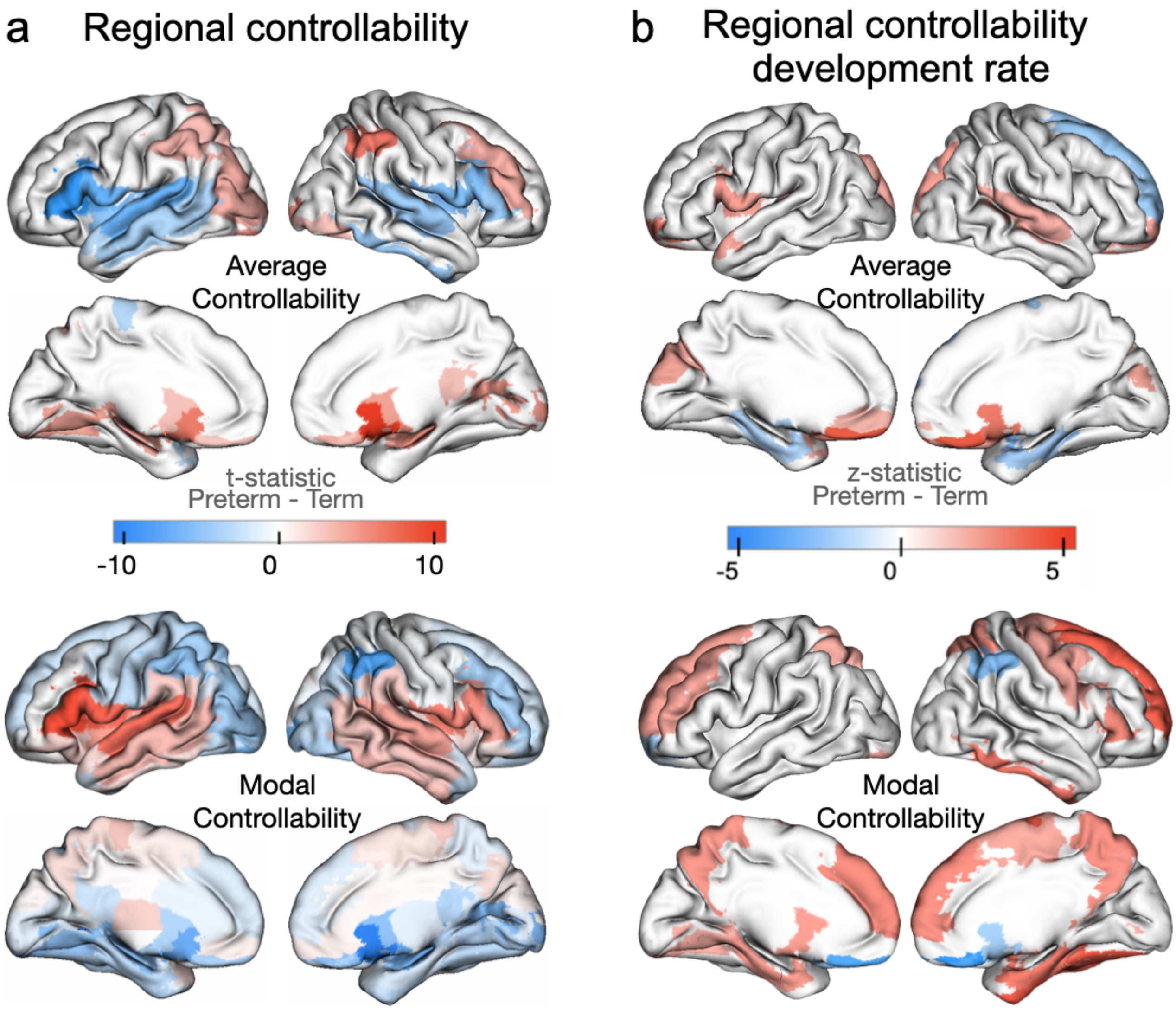

Fig. S5: Group differences in controllability between preterm and term infants with network strength as the additional covariance. (a) Average and modal controllability distribution (controlling for network strength) differences between preterm and term infants (t stats value from the two-sampled two-sided t-test). (b) Average and modal controllability development rate differences (controlling for network strength) between preterm and term infants (z stats value from correlation comparison).

Source data are provided as a Source Data file.

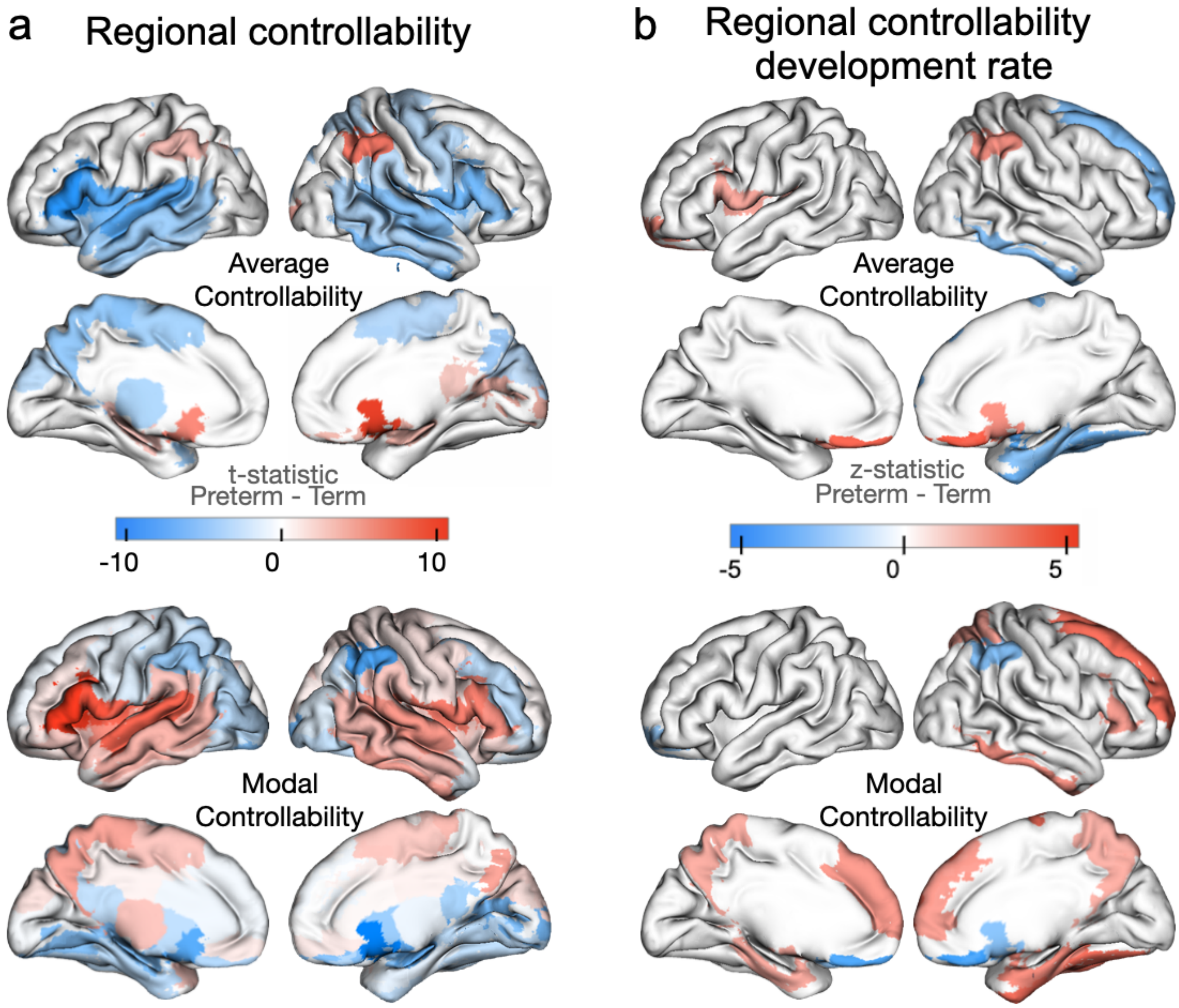

Fig. S6: Group differences in controllability between preterm and term infants with network density as the additional covariance. (a) Average and modal controllability distribution (controlling for network density) differences between preterm and term infants (t stats value from the two-sampled two-sided t-test). (b) Average and modal controllability development rate differences (controlling for network density) between preterm and term infants (z stats value from correlation comparison).

Source data are provided as a Source Data file.

Sex difference in the controllability of the infant brain structural network

Another regular concern about research on infants is whether sex plays a deterministic role, or more specifically, whether the results align in female/male groups. Here, we repeated the analysis in the main text for females and males separately. The whole-brain controllability distribution shows high consistency between the female and male infants for preterm and term groups (Spearman's correlation: $r>0.96$, $p<0.001$) (Table S5). In general, females and males follow similar maturation patterns compared to that of the whole group: average controllability increases with the speed slowing down and modal controllability decreases at a constant pace (Table S5). For the association between regional controllability value and its maturation, similar results are observed for females and males, except the association between regional average controllability and its maturation for males in the term subgroup is not significant (Table S6).

Table S5: Sex consistency in the whole-brain controllability (Spearman's correlation between whole-brain controllability in female and male groups)

	Average Controllability		Modal Controllability	
	r	*p*	*r*	*p*
Preterm at birth	0.96	0	0.96	0
Preterm at TEA	0.98	0	0.98	0
Term	0.99	0	0.99	0

Table S6: Sex difference in controllability maturation (Pearson's correlation between controllability and post-menstrual age)

	Female				Male			
	Average Controllability		Modal Controllability		Average Controllability		Modal Controllability	
	r	*p*	*r*	*p*	*r*	*p*	*r*	*p*
Preterm at birth	0.72	3.8e-6	-0.51	0.0030	0.54	2.7e-4	-0.48	0.0015
Preterm at TEA	0.11	0.57	-0.47	0.0069	0.30	0.06	-0.33	0.033
Term	0.044	0.53	-0.53	2.9e-16	0.04	0.51	-0.51	5.5e-17

Null models for control energy cost

To determine whether the topology of the infant brain structure facilitated transitions to target state of various functional cognitive networks, we built 100 null models for each subject and compared the control energy consumption of real brain structural networks to that of null models using a paired t-test. We found that for both term and preterm infants, the real brain structural networks required less energy to drive the state transitions to every functional network, with t-test results shown in Table S7.

Table S7: Control energy consumption for null models and real brain networks of term and preterm infants (t-stats from paired t-test)

Target State	null term vs term		null preterm vs preterm	
	t	*p*	*t*	*p*
**Visual**	104.14	1.38e-315	51.24	8.08e-58
**Somatomotor**	97.27	6.6e-303	49.32	1.1e-56
**Dorsal Attention**	99.05	2.9e-306	51.50	5.6e-58
**Ventral Attention**	92.26	4.0e-293	49.08	1.6e-56
**Limbic**	95.02	2.0e-302	50.89	1.3e-57
**Frontoparietal**	94.23	5.1e-297	48.92	2.0e-56
**Default Mode**	96.11	1.1e-300	50.02	4.38e-57
**Subcortical**	97.96	3.2e-304	51.33	7.2e-58

Association with cognitive, language, and motor domains of BSID-III scores

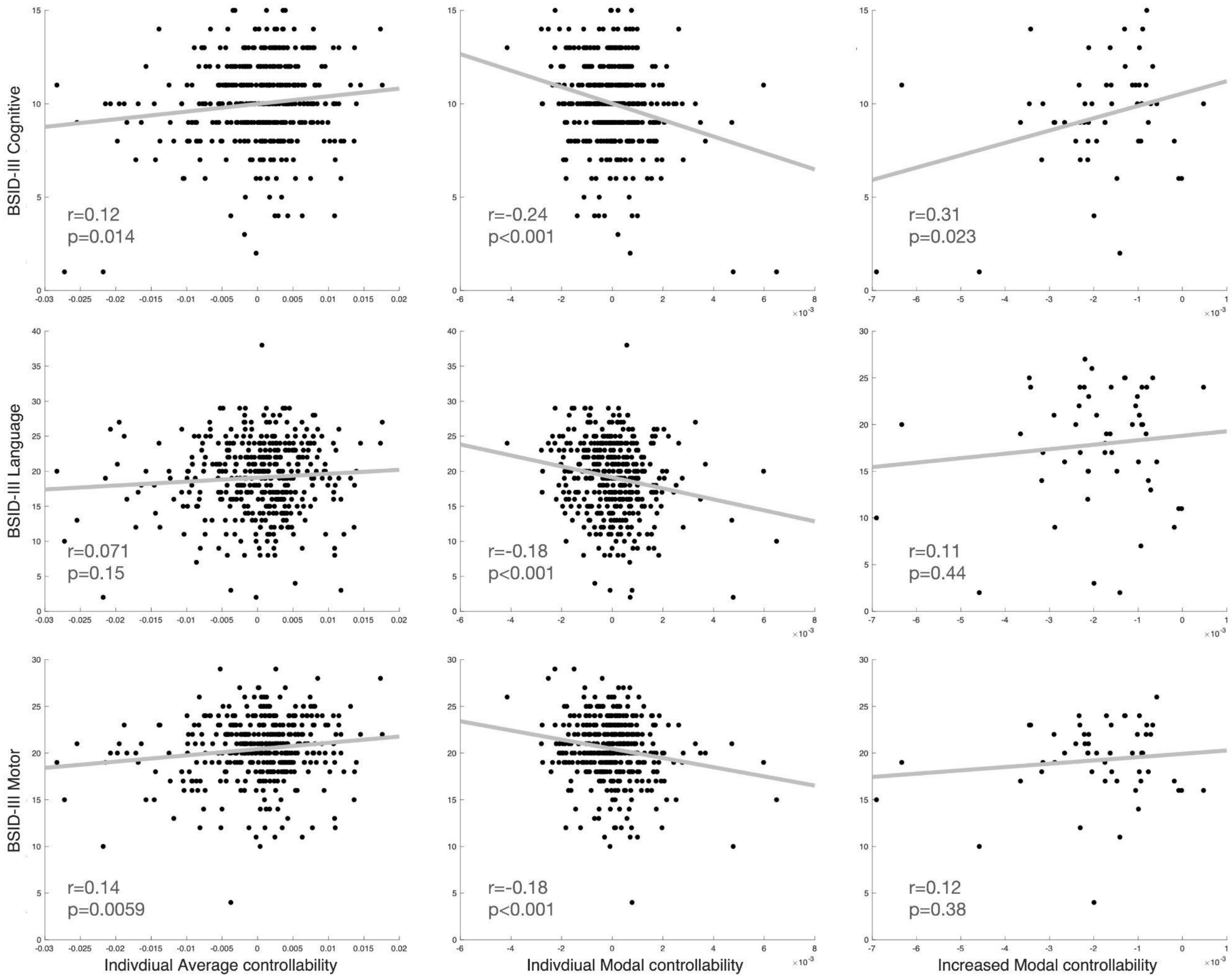

Fig. S7: Scatterplots of controllability at birth and behavioral performances at 18 months old.

Significant Pearson's correlations were observed in BSID-III cognitive scores on the 1st row and individual average controllability (r=0.12, p=0.014) and modal controllability (r=-0.24, p=1.3e-6) on the whole-brain level; Increasing modal controllability for preterm infants from birth to TEA was positively correlated with the BSID-III cognitive scores (r=0.31, p=0.023) (1st row). Individual modal controllability was significantly correlated with BSID-III language on the 2nd row (r=-0.18 p=2.4e-4) but not average controllability(r=0.071, p=0.15) and longitudinal changes in modal controllability (r=0.11, p=0.44). BSID-III motor scores on the 3rd row were significantly associated with both individual average (r=0.14, p=0.0059) and modal controllability (r=-0.18, p=2.5e-4), but not with the longitudinal changes for preterm infants (r=-0.12, p=0.38). p-value is two-sided. Source data are provided as a Source Data file.

Table S8. Neonatal 90 node parcellation's regional names and resting-state networks(RSNs)

Node	Region	RSNs	Node	Region	RSNs
1	Precentral gyrus left	Dorsal Attention	46	Cuneus right	Subcortical
2	Precentral gyrus right	Dorsal Attention	47	Lingual gyrus left	Visual
3	Superior frontal gyrus (dorsal) left	Dorsal Attention	48	Lingual gyrus right	Visual
4	Superior frontal gyrus (dorsal) right	Dorsal Attention	49	Superior occipital gyrus left	Visual
5	Orbitofrontal cortex (superior) left	Frontoparietal	50	Superior occipital gyrus right	Visual
6	Orbitofrontal cortex (superior) right	Frontoparietal	51	Middle occipital gyrus left	Visual
7	Middle frontal gyrus left	Frontoparietal	52	Middle occipital gyrus right	Visual
8	Middle frontal gyrus right	Frontoparietal	53	Inferior occipital gyrus left	Visual
9	Orbitofrontal cortex (middle) left	Frontoparietal	54	Inferior occipital gyrus right	Visual
10	Orbitofrontal cortex (middle) right	Frontoparietal	55	Fusiform gyrus left	Visual
11	Inferior frontal gyrus (opercular) left	Default Mode	56	Fusiform gyrus right	Visual
12	Inferior frontal gyrus (opercular) right	Default Mode	57	Postcentral gyrus left	Visual
13	Inferior frontal gyrus (triangular) left	Somatomotor	58	Postcentral gyrus right	Visual
14	Inferior frontal gyrus (triangular) right	Somatomotor	59	Superior parietal gyrus left	Visual
15	Orbitofrontal cortex (inferior) left	Somatomotor	60	Superior parietal gyrus right	Visual
16	Orbitofrontal cortex (inferior) right	Somatomotor	61	Inferior parietal lobule left	Somatomotor
17	Rolandic operculum left	Limbic	62	Inferior parietal lobule right	Somatomotor
18	Rolandic operculum right	Limbic	63	Supramarginal gyrus left	Dorsal Attention
19	Supplementary motor area left	Default Mode	64	Supramarginal gyrus right	Dorsal Attention
20	Supplementary motor area right	Default Mode	65	Angular gyrus left	Frontoparietal
21	Olfactory left	Default Mode	66	Angular gyrus right	Frontoparietal
22	Olfactory right	Default Mode	67	Precuneus left	Somatomotor
23	Superior frontal gyrus (medial) left	Limbic	68	Precuneus right	Somatomotor

24	Superior frontal gyrus (medial) right	Limbic	69	Paracentral lobule left	Frontoparietal
25	Orbitofrontal cortex (medial) left	Limbic	70	Paracentral lobule right	Frontoparietal
26	Orbitofrontal cortex (medial) right	Limbic	71	Caudate left	Default Mode
27	Rectus gyrus left	Limbic	72	Caudate right	Default Mode
28	Rectus gyrus right	Limbic	73	Putamen left	Somatomotor
29	Insula left	Limbic	74	Putamen right	Somatomotor
30	Insula right	Limbic	75	Pallidum left	Subcortical
31	Anterior cingulate gyrus left	Limbic	76	Pallidum right	Subcortical
32	Anterior cingulate gyrus right	Limbic	77	Thalamus left	Subcortical
33	Middle cingulate gyrus left	Ventral Attention	78	Thalamus right	Subcortical
34	Middle cingulate gyrus right	Ventral Attention	79	Heschl gyrus left	Subcortical
35	Posterior cingulate gyrus left	Default Mode	80	Heschl gyrus right	Subcortical
36	Posterior cingulate gyrus right	Default Mode	81	Superior temporal gyrus left	Subcortical
37	Hippocampus left	Ventral Attention	82	Superior temporal gyrus right	Subcortical
38	Hippocampus right	Ventral Attention	83	Temporal pole (superior) left	Somatomotor
39	ParaHippocampal gyrus left	Default Mode	84	Temporal pole (superior) right	Somatomotor
40	ParaHippocampal gyrus right	Default Mode	85	Middle temporal gyrus left	Somatomotor
41	Amygdala left	Subcortical	86	Middle temporal gyrus right	Somatomotor
42	Amygdala right	Subcortical	87	Temporal pole (middle) left	Limbic
43	Calcarine cortex left	Subcortical	88	Temporal pole (middle) right	Limbic
44	Calcarine cortex right	Subcortical	89	Inferior temporal gyrus left	Default Mode
45	Cuneus left	Subcortical	90	Inferior temporal gyrus right	Default Mode

Supplementary reference

1. Gu, S. *et al.* Controllability of structural brain networks. *Nat. Commun.* **6**, 8414 (2015).
