# Supplementary material for: Network controllability of structural connectomes in the neonatal brain
Source: Nat Commun. 2023 Sep 19;14:5820. doi: 10.1038/s41467-023-41499-w (PMC10509217; doi:10.1038/s41467-023-41499-w)
Supplement: Supplementary file 3 — Reporting Summary [file 41467_2023_41499_MOESM3_ESM.pdf]

nature portfolio

Corresponding author(s): Huili Sun, Dustin Scheinost

Last updated by author(s): 2023/5/3

Reporting Summary

Nature Portfolio wishes to improve the reproducibility of the work that we publish. This form provides structure for consistency and transparency in reporting. For further information on Nature Portfolio policies, see our Editorial Policies and the Editorial Policy Checklist.

Statistics

For all statistical analyses, confirm that the following items are present in the figure legend, table legend, main text, or Methods section.

n/a	Confirmed	
☐	☒	The exact sample size (n) for each experimental group/condition, given as a discrete number and unit of measurement
☐	☒	A statement on whether measurements were taken from distinct samples or whether the same sample was measured repeatedly
☐	☒	The statistical test(s) used AND whether they are one- or two-sided<br>*Only common tests should be described solely by name; describe more complex techniques in the Methods section.*
☐	☒	A description of all covariates tested
☐	☒	A description of any assumptions or corrections, such as tests of normality and adjustment for multiple comparisons
☐	☒	A full description of the statistical parameters including central tendency (e.g. means) or other basic estimates (e.g. regression coefficient) AND variation (e.g. standard deviation) or associated estimates of uncertainty (e.g. confidence intervals)
☐	☒	For null hypothesis testing, the test statistic (e.g. F, t, r) with confidence intervals, effect sizes, degrees of freedom and P value noted<br>*Give P values as exact values whenever suitable.*
☒	☐	For Bayesian analysis, information on the choice of priors and Markov chain Monte Carlo settings
☐	☒	For hierarchical and complex designs, identification of the appropriate level for tests and full reporting of outcomes
☐	☒	Estimates of effect sizes (e.g. Cohen's d, Pearson's r), indicating how they were calculated

Our web collection on statistics for biologists contains articles on many of the points above.

Software and code

Policy information about availability of computer code

Data collection	All data were acquired from the Developing Human Connectome Project (dHCP, http://www.developingconnectome.org/), a large, cross-sectional open science study of infant brain development. The study was approved by the National Research Ethics Service West London committee, and written consent was obtained from participating families before imaging. All scans were collected in the Evelina Newborn Imaging Centre, St Thomas' Hospital, London, UK. Diffusion magnetic resonance imaging (MRI) data and all other MRI data were acquired with a Philips Achieva 3T scanner (Philips Medical Systems, Best, The Netherlands) with a dHCP-customized neonatal imaging system including a 32-channel receive neonatal heal coil (Rapid Biomedical GmbH, Rimpar, DE). Infants were scanned during unsedated sleep after feeding and immobilization in a vacuum-evacuated bag, with hearing protection and physiological monitoring (including pulse oximetry, body temperature, and electrocardiography data) applied during scanning.
Data analysis	Preprocessing code including the FACT algorithm can be found in https://brain.labsolver.org/hcp_d2.html. Network control theory code can be found at https://complexsystemsupenn.com/codedata: (controllability https://complexsystemsupenn.com/s/controllability_code-smb8.zip; control energy https://github.com/ursbraun/network_control_and_dopamine). Custom analysis code is available at https://github.com/huiliii/infant_control.

For manuscripts utilizing custom algorithms or software that are central to the research but not yet described in published literature, software must be made available to editors and reviewers. We strongly encourage code deposition in a community repository (e.g. GitHub). See the Nature Portfolio guidelines for submitting code & software for further information.

Data

Policy information about availability of data

All manuscripts must include a data availability statement. This statement should provide the following information, where applicable:

- Accession codes, unique identifiers, or web links for publicly available datasets
- A description of any restrictions on data availability
- For clinical datasets or third party data, please ensure that the statement adheres to our policy

Raw Data from the Developing Human Connectome Project is publicly available on http://www.developingconnectome.org/data-release/third-data-release and can be downloaded upon request from https://biomedia.github.io/dHCP-release-notes/download.html. The relevant data to generate the figures are provided as Source Data files.

Human research participants

Policy information about studies involving human research participants and Sex and Gender in Research.

Reporting on sex and gender	Sex was determined as biological attributes for participants in the supplementary results. No significant differences were observed between the results in males and females.
Population characteristics	We included 448 term infants (209 female, 239 male) and 73 preterm infants (32 female, 41 male) with longitudinal scans (at birth and TEA) from the second data release of dHCP. Preterm infants were born between 23.57 weeks and 37.00 weeks of gestation and scanned twice. The first was around three weeks after birth, at a mean of 33.73 weeks. The second was at term-equivalent age with a mean of 41.19 weeks. Term infants were born between 37.14 weeks and 42.29 weeks of gestation and scanned between 37.43 weeks and 44.71 weeks. Demographic information is summarized in Table.1.
Recruitment	Research subjects were recruited as part of the developing HUman Connectome Project, approved by the UK National Research Ethics Authority (14/LO/1169). Written consent was obtained from all families. No potential bias in recruitment is present to the current knowledge.
Ethics oversight	The study was approved by the National Research Ethics Service West London committee, and written consent was obtained from participating families before imaging, as noted in http://www.developingconnectome.org/project/. The Yale IRB provided exemption from human subject research.

Note that full information on the approval of the study protocol must also be provided in the manuscript.

Field-specific reporting

Please select the one below that is the best fit for your research. If you are not sure, read the appropriate sections before making your selection.

☒ Life sciences ☐ Behavioural & social sciences ☐ Ecological, evolutionary & environmental sciences

For a reference copy of the document with all sections, see nature.com/documents/nr-reporting-summary-flat.pdf

Life sciences study design

All studies must disclose on these points even when the disclosure is negative.

Sample size	We included all available subjects released from the Developing Human Connectome Project.
Data exclusions	130 repeated scans were excluded before preprocessing. Exclusion criterion during preprocessing was based on neighboring DWI correction: 34 scans were excluded due to their low NDC values identified by a median-value based outlier detector based on QSIPrep recommendations. Lower NDC values reflect reduced data quality, driven by noise and misalignment between dMRI volumes. For preterm infants, only subjects with both scan at birth and scan at term-equivalent age were included in this study in order to conduct group difference analysis. In the end, we included 594 scans (448 scans from 448 term infants and 146 repeated scans from 73 preterm infants) in this study.
Replication	No replication has been included in the current study for the limitation in infant brain data.
Randomization	Not relevant to the current study because the group allocation (preterm vs term) is determined by the demographic characteristics of each participant.
Blinding	Investigators were blinded to group allocation during data collection and analysis.

Reporting for specific materials, systems and methods

We require information from authors about some types of materials, experimental systems and methods used in many studies. Here, indicate whether each material, system or method listed is relevant to your study. If you are not sure if a list item applies to your research, read the appropriate section before selecting a response.

Materials & experimental systems

n/a	Involved in the study
x	Antibodies
x	Eukaryotic cell lines
x	Palaeontology and archaeology
x	Animals and other organisms
x	Clinical data
x	Dual use research of concern

Methods

n/a	Involved in the study
x	ChIP-seq
x	Flow cytometry
	x MRI-based neuroimaging

Magnetic resonance imaging

Experimental design

Design type: Diffusion tensor image (DTI)

Design specifications: Infants were scanned during unsedated sleep after feeding and immobilization in a vacuum-evacuated bag, with hearing protection and physiological monitoring (including pulse oximetry, body temperature, and electrocardiography data) applied during scanning.

Behavioral performance measures: Follow-up assessments on neurodevelopmental and neurocognitive testing were performed at 18 months for 79% subjects scanned at birth with Bayley-III test to measure the cognitive, language and motor abilities during their toddler hood.

Acquisition

Imaging type(s): Structural, Diffusion

Field strength: 3T

Sequence & imaging parameters: T2-weighted images were obtained using a Turbo spin echo sequence (TR=12s, TE=156ms, SENSE factor 2.11 (axial) and 2.54 (sagittal) with overlapping slices (resolution = 0.8x0.8x1.6 mm3).

Area of acquisition: Whole-brain

Diffusion MRI: [x] Used [ ] Not used

Parameters: Diffusion-weighted imaging (DWI) was obtained in 300 directions (TR=3.8s, TE=90ms, SENSE factor 1.2, multiband factor 4, and resolution 1.5x1.5x3mm3 with 1.5 mm slice overlap) with b-values of 400s/mm2, 1000s/mm2 and 2600 s/mm2 spherically distributed in 64, 88 and 128 directions respectively using interleaved phase encoding.

Preprocessing

Preprocessing software: Diffusion MRI was reconstructed at an in-plane resolution of 1.5mm and slice thickness of 1.5mm with the diffusion SHARD pipeline(https://biomedia.github.io/dHCP-release-notes/dwi-shard.html). The whole-brain fiber tracking was conducted with DSI-studio (http://dsi-studio.labsolver.org/) with the quantitative anisotropy (QA) as the termination threshold. The tracking parameters were set as the angular cutoff of 60 degree, step size of 1.0mm, minimum length of 30 mm, and maximum length of 300 mm. The whole-brain fiber tracking process was performed with the FACT algorithm until 1,000,000 streamlines were reconstructed for each individual.

Normalization: Data of infant brains were not normalized to standard space, but processed in the native DWI space with T2-weighted images.

Normalization template: Data were not normalized.

Noise and artifact removal: Images were denoised and corrected for motion, eddy current, Gibbs ringing, and susceptibility artifact with the diffusion SHARD pipeline(https://biomedia.github.io/dHCP-release-notes/dwi-shard.html) and checked by neighboring DWI correction (NDC).

Volume censoring: No volume censoring applies for DTI data.

Statistical modeling & inference

Model type and settings	Mass univariate linear model with FDR
Effect(s) tested	Linear models were used to test DTI-related measurements in this study.

Specify type of analysis: ☒ Whole brain ☐ ROI-based ☐ Both

Statistic type for inference (See Eklund et al. 2016)	Connectome-wise inference
Correction	The Benjamini-Hochberg false discovery rate (FDR) method was used to correct for multiple comparisons.

Models & analysis

n/a	Involved in the study
☒	☐ Functional and/or effective connectivity
☐	☒ Graph analysis
☒	☐ Multivariate modeling or predictive analysis

Graph analysis	Based on this network control theory framework, two diagnostics of controllability that describe the ability to drive the network with different types of transitions as patterns of regional activity were investigated here: average controllability to measure the ability to drive nearby brain state transition and modal controllability to estimate that of distant brain state transition on the brain energy landscape. Whole-brain controllability was calculated as the mean controllability across all brain regions for each subject, and regional controllability was calculated as the mean controllability across all subjects on the group level.
